# Synthesis and Characterization of Silver-Gold Bimetallic Nanoparticles for Random Lasing

**DOI:** 10.3390/nano12040607

**Published:** 2022-02-11

**Authors:** Wan Zakiah Wan Ismail, Judith M. Dawes

**Affiliations:** 1 Advanced Devices and System, Faculty of Engineering and Built Environment, Universiti Sains Islam Malaysia, Nilai 71800, Negeri Sembilan, Malaysia; 2 MQ Photonics, Faculty of Science and Engineering, Macquarie University, Sydney 2109, Australia; judith.dawes@mq.edu.au

**Keywords:** bimetallic, nanomaterial, localized surface plasmon effects, surface roughness, and random lasers

## Abstract

We developed rough silver-gold bimetallic nanoparticles for random lasing. Silver nanoparticles were synthesized based on a citrate-reduction method and the gold (III) chloride trihydrate was added to produce bimetallic nanoparticles. Gold atoms were deposited on the surface of the silver (Ag) through galvanic replacement reactions after the solution was stored at room temperature. Sample characterization and a spectrometry experiment were performed where bimetallic nanoparticles with nanogaps and the extinction of the nanoparticles were observed. The aim of this research is to synthesize nanoparticles for random dye laser in a weakly scattering regime. The novel bimetallic nanoparticles were added to Rhodamine 640 solution to produce random lasing. We found that random dye laser with bimetallic nanoparticles produced spectral narrowing and lasing threshold compared to random dye laser with silver nanoparticles. We attribute that to the localized surface plasmon effects which increase local electromagnetic field to provide sufficient optical gain for random lasing. The rough surface of bimetallic nanoparticles also contributes to the properties of random lasing. Thus, we suggest that the rough bimetallic nanoparticles can be used to develop random lasers.

## 1. Introduction

Nowadays, research on metal nanoparticles is broad, comprising of many fields such as sensing [1], laser bioprinting [2] and random lasers [3,4,5]. There are two common techniques used to prepare metallic nanoparticles; (1) Reduction of the metal cations and (2) Disassembling larger objects by grinding or laser ablation [6]. Both methods have advantages and disadvantages. The first method requires a suitable soluble source of metals and the processes of nucleation and crystal growth are complex, whereas the second method allows for greater control of the metal elements but requires high costs in terms of the samples and laser sources [6]. Bimetallic nanoparticles were used to produce random lasers [5] where gold-silver bimetallic nanowires were fabricated to provide strong feedback. In this random laser, Rhodamine 6G (Rh6G) dye was used because it can absorb maximum excitation light.

Random lasers are lasers which need multiple light scattering for feedback and optical gain for light amplification. The laser can be used as a light source for optical tomography, biosensing and spectroscopy. Properties of random lasers consist of lasing threshold, emission linewidth, emission intensity and polarization. The properties can be influenced by the surface roughness of scatterers [5,7,8,9]. Improved feedback can be provided by scattering induced by rough micron-scale grooves [8] or nano-rubbing induced cavities [7]. Random lasing can be observed in dye solution with metal nanowires in which nanowires act as nano-antennas to enhance plasmonic effects [10] and the emission wavelength range can be extended by introducing roughness to the silver nanowires [9]. Surface roughness affects the plasmonic properties of the metal nanoparticles by significantly enhancing local fields and plasmonic effects compared to smooth metal nanoparticles. This leads to peak-broadening and red-shifting of the single particle-plasmon resonances [11]. J. Liao et al. [12] proved that the tunability of both intensity and wavelength of the localized surface plasmon resonance (LSPR) can be influenced by the thickness of the bimetallic nanoparticles’ film and the number of pulse shots.

Besides that, the surface plasmon resonance frequency was able to be tuned to the desired spectral range by varying the thickness of the outer metal shell, the thickness and dielectric constant of the intermediate dielectric medium, the geometric shape, and the aspect ratio of the three-layered bimetallic nanoparticles [13]. Random lasing is greatly influenced by the overlapping of surface plasmon resonance of metallic nanoparticles and the emission spectrum of the dye medium [13,14]. This situation does not only increase the dye excitation and energy transfer, but also affect the dye emission, leading to local field enhancement [13,14,15]. Thus, overlapping of these elements can influence the properties of random lasing.

In this work, we aim to create reliable bimetallic nanoparticles using citrate-reduction method [16] and galvanic replacement reactions [17] for random lasing. In a one-step reduction method, sodium citrate and various quantities of tannic acid were used in the growth process to produce mono-dispersed spherical silver nanoparticles [16]. Galvanic reactions occurred when aqueous solution of AuCl_4_ or AuCl_2_ was titrated into aqueous suspension of silver nanoparticles [17]. Chemical reduction method is chosen in this study due to lower cost compared to laser ablation technique. Bimetallic nanoparticles are formed when gold atoms are deposited on the surface of silver nanoparticles. The nanoparticles were added in a dye solution (Rhodamine 640) and excited by a Nd:YAG laser. Random lasing with a narrow emission linewidth, ~3 nm can be observed. Most previous studies on random lasers for weakly scattering regime used Rhodamine 6G (Rh6G) [3,4,5,18,19] instead of Rhodamine 640 (Rh640) due to higher absorption of excitation light at 530 nm. Here, we prove that random lasing can be realized in Rh640 solution using rough silver-gold bimetallic nanoparticles in weakly scattering regime. Rh640 does not absorb the excitation light as good as Rh6G but bimetallic nanoparticles can compensate that by enhancing optical gain for more light amplification. Gold is chosen to produce silver-gold bimetallic nanoparticles due to good LSP effect. Thus, random lasing can be attributed to the surface roughness and LSPs induced by bimetallic nanoparticles. The nanoparticles scatter the light, which then affecting the gain and feedback of random lasers respectively. Metallic nanoparticles that are excited by light can induce LSP, resulting from the confinement of surface plasmons in the nanoparticles. The conduction electrons oscillate coherently because of oscillating electric field. Thus, LSP can give maximum absorption at the plasmon resonant frequency and enhanced electric fields near the surface of the particles [5,20].

## 2. Synthesis, Characterization and Optical Measurement

### 2.1. Synthesis of Silver Nanoparticles

Bimetallic nanoparticles consist of silver nanoparticles with gold deposited on the surface of the silver nanoparticles. The citrate-reduction method was used to produce silver nanoparticles [16,21]. Silver nitrate aqueous solution was prepared by dissolving 0.009 g of silver nitrate (AgNO_3_) (Sigma Aldrich, Burlington, MA, USA) into 49 mL of deionized water. The solution was vigorously stirred (1000 rpm) and heated on a hot plate to boiling (100 °C), confirmed by a thermometer.

Then, 1 mL of freshly prepared trisodium citrate (34 mM) (Sigma Aldrich, Burlington, MA, USA) was added dropwise within 1 min to the boiling silver nitrate aqueous solution. The dropwise-addition is important to produce small-size silver nanoparticles [21]. The solution started to change colour from transparent to light yellow after adding the trisodium citrate. Then, it changed to dark yellow/orange after 6 min and finally, to pale green within 15 min. The reaction solution was cooled at room temperature before centrifugation (by Eppendorf Mini Spin, Sigma Aldrich, Burlington, MA, USA) using 2.7 krpm for 1 h to remove larger silver colloids. The supernatant silver colloid was taken and the size of the colloid was measured using the transmission electron microscopy (TEM). Then, the silver colloids were washed and dispersed in deionized water for further experiments.

### 2.2. Synthesis of Rough Silver-Gold Bimetallic Nanoparticles

**Figure 1:**
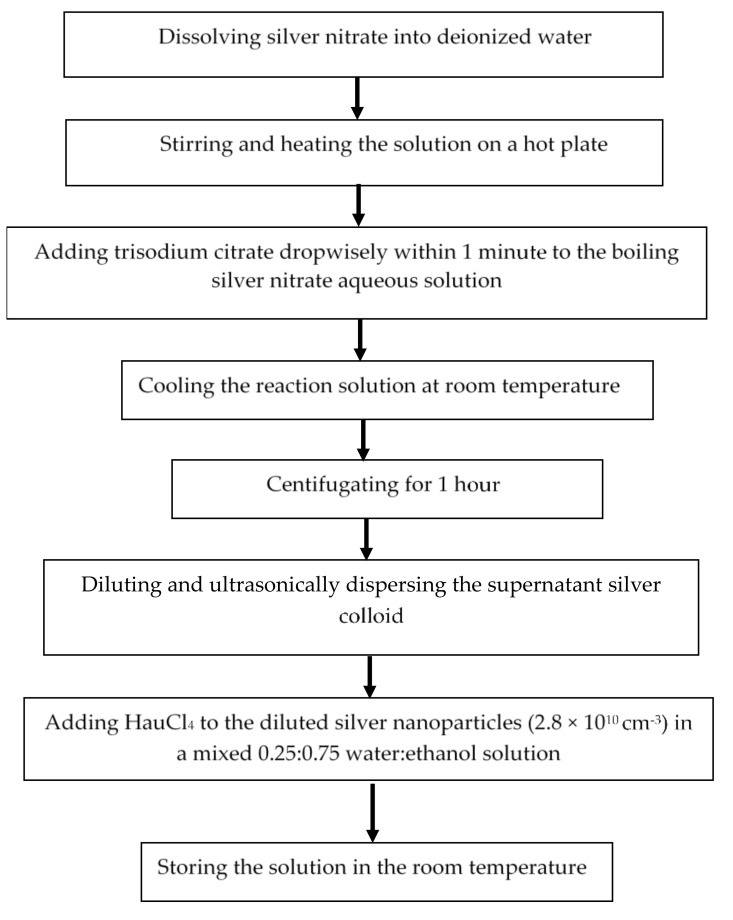
The synthesis flow of the bimetallic nanoparticles.

To prepare bimetallic nanoparticles, the as-prepared silver nanoparticle solution was diluted (4×) and ultrasonically dispersed to prevent aggregation. Gold (III) chloride trihydrate (HAuCl4) (4.8 mM) (Sigma Aldrich, Burlington, MA, USA) varied from 40 μL to 120 μL was added to the diluted silver nanoparticles in a mixed 0.25:0.75 water:ethanol solution and the solution was stored at room temperature overnight (12 h) to ensure gold (Au) atoms were deposited on the surface of the silver (Ag) through galvanic replacement reactions [17,22]. The synthesis flow of the bimetallic nanoparticles can also be found in Figure 1.

In galvanic replacement reaction, when a metal ion with higher reduction potential is in contact with another metal with lower reduction potential in an electrolyte, an electrochemical reaction occurs that leads to the preferential corrosion of the second metal. When the metal salt solution was added, HAuCl_4_ in silver colloid, the metal (Au) was deposited around silver nanoparticles. Thus, a silver nanoparticle template was concurrently removed from the inside out through gaps in the deposited layer [22]. Porous and rough silver-gold bimetallic nanoparticles can be produced from galvanic replacement reaction.

### 2.3. Characterization of Bimetallic Nanoparticles Using Energy Dispersive X-ray Analysis (EDX)

Increasing amount of HAuCl_4_ can increase the weight of Au(gold) in the sample [17]. Table 1 shows percentage of gold (weight %) in the silver-gold bimetallic nanoparticles by varying amount of HAuCl_4_ based on energy dispersive X-ray analysis (EDX). The EDX is used to identify the silver and gold elements of the nanoparticles based on an X-ray method.

### 2.4. Characterization of Bimetallic Nanoparticles Using Transmission Electron Microscopy (TEM) and Zetasizer

**Figure 2:**
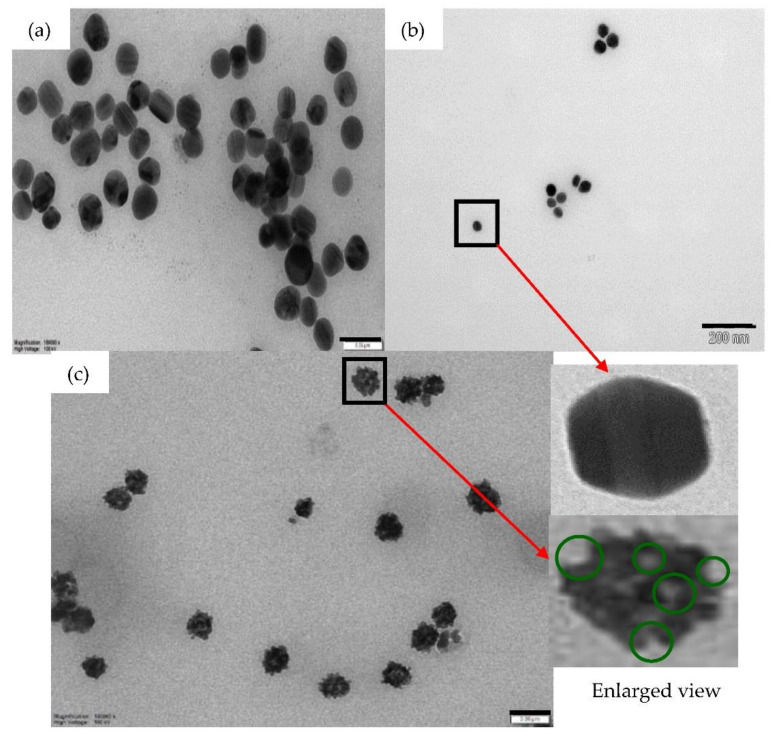
TEM images of silver nanoparticles (1.1 × 10^11^ cm^−3^) (**a**) before centrifugation with a scale bar of 100 nm and (**b**) after centrifugation for 1 h (10× dilution) with a scale bar 200 nm. From that Figure, the size of nanoparticles was estimated as ~30 ± 2 nm. (**c**) TEM images of rough silver-gold bimetallic nanoparticles (2.8 × 10^10^ cm^−3^) with a scale bar of 50 nm. The enlarged view of a silver nanoparticle and a rough bimetallic nanoparticle shown are also shown in the Figure. The green circles show the nanogaps. 40 μL of HAuCl_4_ was used in the sample shown in Figure 2c.

The TEM images of the silver colloids are shown in Figure 2a. Slight aggregation is observed since the silver colloids are not diluted. The average diameter of the silver nanoparticles is ~30 ± 2 nm from TEM measurement. The particle concentration was estimated by drying and weighing the dried samples, which gives 2.2 × 10^−5^ g/cm^3^ or 1.1 × 10^11^ cm^−3^. To estimate the particle concentration, 1 mL of silver colloids was washed with deionized water and centrifuged using the highest speed, 14 krpm. The remaining sediment was dried in vacuum and weighed to estimate the amount of silver nanoparticles.

Figure 2b shows the TEM images of the silver colloids after centrifugation with the estimated average silver diameter, 30 ± 2 nm. Figure 2c shows the TEM image of the rough bimetallic nanoparticles. The enlarged images of silver and bimetallic nanoparticles are shown in Figure 2 where we observe a smooth surface of silver nanoparticles and a rough surface of silver-gold bimetallic nanoparticles. Adding HAuCl_4_ to the silver nanoparticles can produce rough bimetallic nanoparticle as the gold atoms attach randomly to the silver nanoparticles. Nanogaps can also be observed at the rough surface of the bimetallic nanoparticles (shown by the green circles). Rough surface and nanogaps can be attributed to the galvanic reaction process [17,22]. The average diameter of bimetallic nanoparticles was estimated as ~32 ± 2 nm and the concentration of bimetallic nanoparticles was estimated as ~2.8 × 10^10^ cm^−3^.

**Figure 3:**
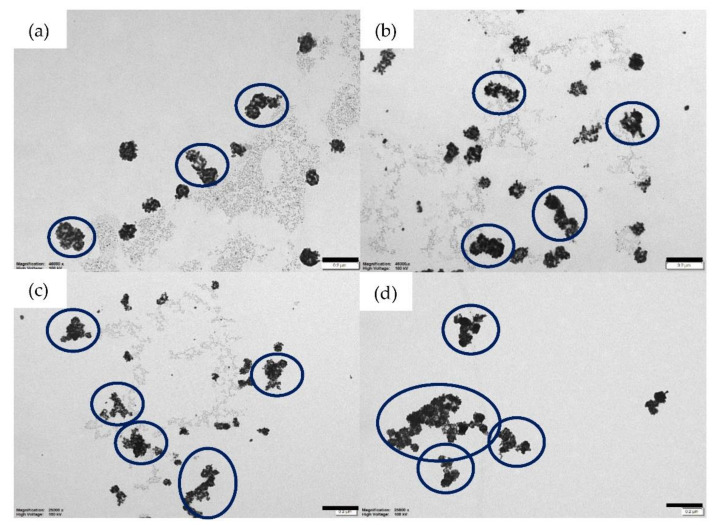
TEM images of bimetallic nanoparticles with various amount of HAuCl_4_; (**a**) 60 μL, (**b**) 80 μL, (**c**) 100 μL and (**d**) 120 μL with a scale bar of 100 nm. The aggregation is shown by blue circles. The highest aggregation of nanoparticles occurs after adding 120 μL of HAuCl_4_ to the silver nanoparticles.

Increasing the amount of HAuCl_4_ does not only increase the amount of gold but also induces aggregation, shown in Figure 3. Slight aggregation appears when 60 μL and 80 μL of HAuCl_4_ were added separately (Figure 3a,b). Some nanoparticles clump and produce larger nanoparticles. More aggregation is observed after adding 100 μL of HAuCl_4_ (Figure 3c). The largest aggregation of nanoparticles occurs when 120 μL of HAuCl_4_ was added to the silver nanoparticles (Figure 3d). Aggregation of silver-gold nanoparticles can reduce scattering, one of the important factors to develop random lasers. Thus, we applied silver-gold nanoparticles that were titrated with 40 μL of HAuCl_4_ for the random laser experiment.

**Figure 4:**
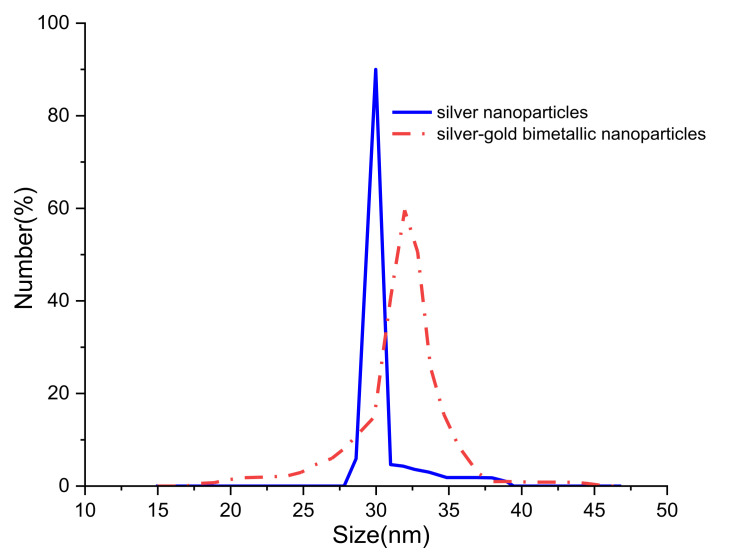
Size distribution of silver nanoparticles and silver-gold nanoparticles. 40 μL of HAuCl_4_ was used in the sample.

Besides TEM, Zetasizer (Malvern Instrument, Malvern, UK) is also used to observe the size distribution of silver-gold bimetallic nanoparticles. Figure 4 shows the size distribution of silver nanoparticles and silver-gold bimetallic nanoparticles where the estimated size of the nanoparticles is similar with TEM method. Uniform size of silver nanoparticles and slight larger size of silver-gold nanoparticles are observed. We attribute that to the effect of titrating HAuCl_4_ inside the silver nanoparticles solution.

The scattering mean-free paths of silver and bimetallic nanoparticles, *l_s_* are estimated as ~446 cm and ~298 cm, based on *l_s_* = 1/(*ρσ_s_*) [4,23,24,25,26] where *ρ* and *σ_s_* refer to the number density of scatterers and scattering cross section, respectively. *σ_s_* for silver and rough bimetallic nanoparticles are estimated as ~8 × 10^−14^ cm^2^ and ~1.2 × 10^−13^ cm^2^ at the optical wavelength of λ~600 nm calculated from Mie theory [25,27,28,29,30,31]. As *l_s_* >> *L* (sample size ~3 mm), the random lasers are in the weakly scattering regime [5,32].

### 2.5. Optical Measurement

**Figure 5:**
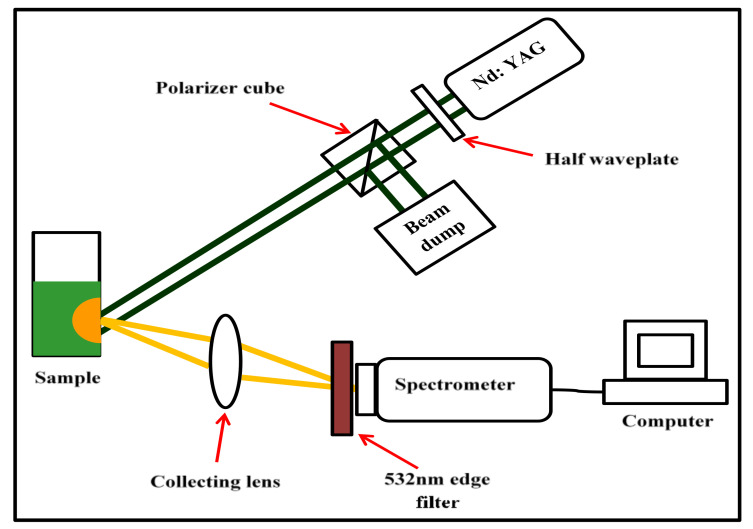
Random laser measurement.

The absorption and fluorescence spectra were measured using a Cary spectrophotometer (Varian, Belrose, Australia) and Fluorolog (Horiba Jobin Yvon, Middlesex, NJ, USA) spectrofluorometer. For random laser measurements (Figure 5), the samples were excited with a Q-switched, frequency-doubled Nd:YAG laser (532 nm, 10 Hz, 4 ns) with a 3 mm diameter excitation spot size at the sample (*L*) at an angle of 45° to the normal to the front face of the cuvette and the emission light was collected from the front face of the cuvette at 30° to the normal by a lens (*f* = 5 cm) and measured by a fibre-coupled spectrometer (Ocean Optics Spectrometer (USB2000+UV-VS-ES) with a resolution ~1 nm). A thin teflon sheet inside the cuvette prevented back-reflection from the cuvette’s faces and a 532 nm edge filter blocked residual pump light from the spectrometer. Rhodamine 640 (1 × 10^−4^ M) was added to the rough bimetallic nanoparticle solution in a quartz cuvette (1 cm × 1 cm) for random laser studies.

## 3. Results in Optics and Discussion

### 3.1. Absorption and Fluorescence Spectra of Samples

**Figure 6:**
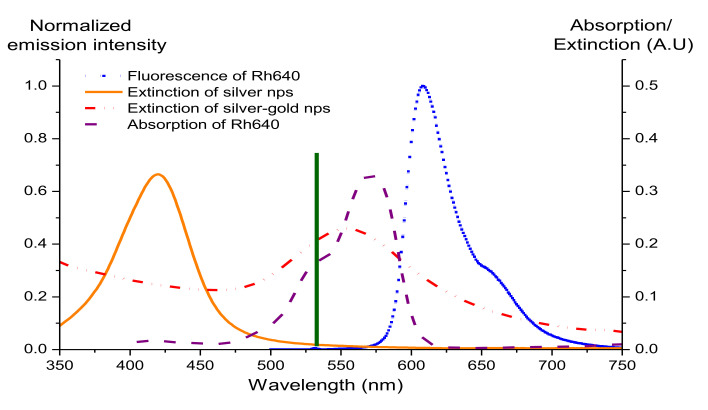
Extinction spectra of silver and bimetallic nanoparticles (nps) and absorption and fluorescence spectrum of Rhodamine 640: Extinction spectrum of silver (orange curve); extinction spectrum of rough bimetallic nanoparticles (red dash dot curve); absorption of Rhodamine 640 (purple dash curve) and fluorescence spectrum of Rhodamine 640 (dark blue dot curve). The vertical line (dark green) indicates the pump laser wavelength. Bimetallic nanoparticles were prepared by adding 40 μL of HAuCl_4_.

Figure 6 compares the extinction spectra of silver and silver-gold bimetallic nanoparticles and the absorption and fluorescence spectra of Rhodamine 640. When Au atoms are deposited on the surface of the silver, the silver plasmon resonance peak shifts from 410 nm (orange curve) to 560 nm and broadens (red dash dot), leading to a more effective overlap of the spectra of bimetallic nanoparticles with the pump laser wavelength, absorption, and fluorescence of Rhodamine 640 (Rh640). The overlap may result in coupling between metal and fluorophores and hence localization of the pump light [33].

### 3.2. Random Laser Properties

**Figure 7:**
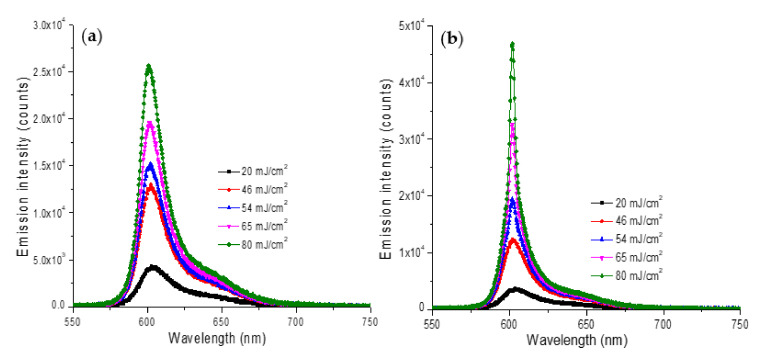
Emission spectra of (**a**) Rh640 (1 × 10^−4^ M)/silver and (**b**) Rh640 (1 × 10^−4^ M)/rough bimetallic random lasers.

Figure 7a,b present the emission spectra of Rh640 random lasers with silver nanoparticles and with bimetallic nanoparticles, respectively. We observe distinctive spectral narrowing for the Rh640/bimetallic random lasers (Figure 7b).

**Figure 8:**
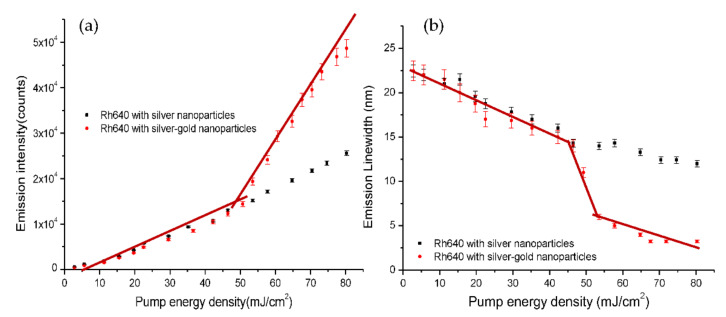
(**a**) Emission peak intensity and (**b**) Emission linewidth at 75% of emission peak intensity as a function of pump energy density for Rh640 (1 × 10^−4^ M)/silver and Rh640/bimetallic random lasers. The lasing threshold is estimated when the emission peak intensity increases substantially with the pump level or when the emission linewidth drops significantly as indicated by the brown line. Error bars show the fluctuation of the emission intensity and linewidth for ten readings from Ocean Optics Spectrometers.

Rh640/bimetallic random lasers exhibit a lasing threshold at ~53 mJ/cm^2^, as estimated from an increase of the emission peak intensity and a decrease of the emission linewidth (75% of emission peak intensity) as shown in Figure 8. Meanwhile, Rh640/silver random lasers only produce a linear increase of the emission peak intensity and a decrease of the emission linewidth (Figure 8). The emission linewidth gradually decreases from 23 nm to 15 nm for Rh640/silver random lasers while the emission linewidth decreases from 23 nm to 5 nm for Rh640/bimetallic random lasers. We observe an increase of the emission peak intensity and a step decrease of the emission linewidth with the pump level for Rh640/bimetallic random lasers. A sharp reduction of the emission linewidth indicates the onset of the stimulated emission [34]. Here, we estimate the lasing threshold from ~52 mJ/cm^2^ to 55 mJ/cm^2^ when the emission peak intensity starts to increase substantially and emission linewidth decreases from 12 nm to 5 nm (Figure 8). Rh640/bimetallic random lasers exhibit a clear threshold with a decrease of emission linewidth due to the LSP effect and the rough surface of bimetallic nanoparticles.

Spectral narrowing and lasing threshold are evidence of random lasing with incoherent feedback [32,35]. The Rh640/bimetallic random lasers exhibit these characteristics due to two factors: (1) plasmon effects and (2) surface roughness.

(1) Silver nanoparticles have a surface plasmon band within range 350 nm to 500 nm with a plasmon peak at ~410 nm (Figure 6) and this leads to almost negligible plasmon effects on the fluorescence spectrum of Rhodamine 640 whereas the plasmon resonance peak of the bimetallic nanoparticles red-shifts and broadens. The scattering mean-free path of the silver nanoparticle solution, *l_s_*~446 cm at λ~600 nm indicates the weakly scattering regime [5,32] with less scattering. With insufficient scattering and almost negligible plasmon effects to increase the gain, no random lasing is observed for the Rh640/silver nanoparticles. In contrast, the bimetallic nanoparticles have a plasmon band in the visible range which overlaps effectively with the absorption and fluorescence of Rh640 and gives plasmonic enhancement to the Rh640/bimetallic nanoparticle random lasers. Though the bimetallic nanoparticles are in the weakly scattering regime (*l_s_*~298 nm at λ~600 nm) [5,32], the plasmon effects can compensate for the weak scattering and produce effective random lasing.

(2) Silver nanoparticles with smooth surfaces (Figure 2b) are compared to the bimetallic nanoparticles with rough, porous surfaces (Figure 2c). The roughness of the bimetallic nanoparticles is due to gold atoms attached to the silver surface. The dye molecules can be concentrated near the nanogaps and the nanogaps form energy “hotspot” which increase the local electromagnetic field [5]. The increase of electromagnetic field may enhance the optical gain that is important for light amplification.

Studies of sensors and light sources based on random lasers are developing very fast. Recent research includes the usage of silver nanoparticles and polymer film on fiber facet [36], whispering-gallery mode lasers [37], fabricating random laser-based humidity sensor by using polymer membrane doped with silver nanoparticles [38] and tunable coherent light source based on metal halide perovskites [39]. Thus, we believe that this research is useful for studies of sensing and light sources due to its simple experimental set up, structure and flexible design. Figure 3 shows that we can control the shape and size of nanoparticles by varying amount of HAuCl_4_. However, further studies are needed to gain better control of random laser properties.

## 4. Conclusions

In conclusion, rough silver-gold bimetallic nanoparticles were created for random laser. The nanoparticles consist of silver nanoparticles with gold atoms deposited on the surface of the silver (Ag) through galvanic replacement reactions. Silver nanoparticles were synthesized using citrate-reduction method and gold (III) chloride trihydrate was added result in roughness and nanogaps on the surface of silver nanoparticles. Characterization was done using TEM where the TEM image of silver nanoparticles and rough bimetallic nanoparticles can be compared. EDX and Zetasizer were also applied to observe the percentage of gold on silver nanoparticles and the size distribution of nanoparticles, respectively. The proposed rough bimetallic nanoparticles in Rh640 solution show a random lasing threshold with a higher emission intensity and a narrower emission linewidth compared to silver nanoparticles in Rh640 solution which is due to plasmon effects and surface roughness. The electromagnetic field can be enhanced when the dye molecules concentrate near the metal surface. All samples are in the weakly scattering regime. Thus, we attribute the spectral narrowing to surface plasmon effects, where the local electric field is enhanced near the surface of silver nanoparticles, leading to enhanced gain in the system. The rough surfaces with nanogaps also can trap dye molecules to increase the electromagnetic field that eventually increase the gain. The enhanced gain can compensate for the reduced scattering that occurs in the weakly scattering regime, to produce better properties of random lasers. In future, laser sources and sensing based on random laser can be developed using the proposed bimetallic nanoparticles.

## Figures and Tables

**Table 1:** The percentage of gold (weight %) in the silver-gold nanoparticles by titrating various amount of HAuCl_4_ based on energy dispersive X-ray analysis (EDX).

Amount of HAuCl_4_ (μL)	Au Composition (wt %)
40 μL	15
60 μL	28
80 μL	35
100 μL	46
120 μL	71

## Data Availability

All data are reported in this article.
